# Supplementary material for: Convergent Mutations and Single Nucleotide Variants in Mitochondrial Genomes of Modern Humans and Neanderthals
Source: Int J Mol Sci. 2024 Mar 28;25(7):3785. doi: 10.3390/ijms25073785 (PMC11012180; doi:10.3390/ijms25073785)
Supplement: Supplementary file 1 [file ijms-25-03785-s001.zip › ijms-2866656-supplementary.pdf]

Supplementary Table S1. GenBank accession numbers of Neanderthal mitogenome sequences used in this study.

H. sapiens neanderthalensis	**GenBank accession**
Vindija 33.16	AM948965
Vindija 33.17	KJ533544
Vindija 33.19	KJ533545
Vindija 33.25	FM865410
Altai Neandertal	KC879692
Feldhofer 1	FM865407
Feldhofer 2	FM865408
El Sidron 1253	FM865409
Mezmaskaya 1	FM865411

Supplementary Table S2. GenBank accession numbers of archaic AMH mitogenome sequences used in this study.

Archaic AMH	GenBank/ENA*	Archaic AMH	GenBank/ENA*
Doni Vestonice 14	KC521458	Hohle Fels 79	KU534962
Fumane 2	KP718913	Goyet Q-2	KU534963
Kostenki 14	FN600416*	Hohle Fels 49	KU534964
Oberkassel 98	KC521457	Goyet Q56-16	KU534965
Tianyuan	KC417443	Goyet Q53-1	KU534966
Ust-Ishim	PRJEB6622*	Goyet Q376-19	KU534967
Brillenhohle	KU534947	Paglicci 108	KU534968
Goyet Q55-2	KU534948	Cioclovina 1	KU534969
Dolni Vestonice 16	KU534949	Dolni Vestonice 43	KU534970
Paglicci 71	KU534950	Rochedane	KU534971
La Rochette	KU534951	Iboussieres 39	KU534972
Goyet Q116-1	KU534952	Bockstein	KU534973
Goyet Q376-3	KU534953	Ofnet	KU534974
Felsdach	KU534954	Cuiry Les Chaudardes 1	KU534975
Goyet 2878-21	KU534955	Iboussieres 31-2	KU534976
Paglicci 133	KU534956	Berry Au Bac 1	KU534977
Rigney 1	KU534957	Ranchot88	KU534978
Les Closeaux 3	KU534958	Hohlenstein Stadel	KU534979
Mareui Les Meaux 1	KU534959	Falkenstein	KU534980
Burkhardtshohle	KU534960	Iboussieres 25-1	KU534981
Hohle Fels 10	KU534961		

Supplementary Table S3. GenBank accession numbers of present-day human mitochondrial haplogroup sequences used in this study.

Haplogroup	GenBank	Haplogroup	GenBank
L0a1b1	AF381988	S1	AF346963
L1c3a	AF381992	Q1	AY289090
L2a1f	AY195776	Z1	AY519493
L3d3b	AF381998	O	AY289059
L4a1	FJ460531	Y1	KF540727
L5a1a	DQ341060	R0a	JX153281
L6a	EU092773	R0a1a3	GU592021
N1b1a3	AY195756	R1a	KC985147
N9a1	HM589048	I1	JQ245776
N2a	JF904935	W1	EU558696
A	AP013225	X1a	EU600318
B2	EF648602	X3	EF177437
F1a1	NA17963	U1a1d	EF692533
M29a	DQ137407	U2c	AY714010
M2b	EU443512	U6a7a2	AF382008
M3b	FJ383523	K	AF382005
M8a1	KF148510	K1	EU073969
M9a	HM346891	K2a2a	EU327986
M20	JX289112	H1a1	EU007858
G1a1	HM460792	H3	EU150187
E1	KF540505	H15	KC911292
D4	JQ704974	J1c	EU547187
C1a	EU007858	J2a2a	EF660967
C4	FJ951604	T1	JQ797976
C7	FJ951594	V1a	JQ702026
P2	AY289088	V2	JQ703647

Supplementary Table S4. Complete list of positions where present-day human mitogenomes are identical with Neanderthals.

Position	Modern haplogroups	Neanderthals
146	L2a1f, L6a, K, K2a2a, R0a1a3, X1a, X3, Y1, M3b, N1b1a3, Q1	Altai, El Sidron 1253, Feldhofer 1, Feldhofer 2, Vindija 33.16, Vindija 33.17, Vindija 33.19, Vindija 33.25
152	L1c3a, L2a1f, L3d3b, L6a, I1, K1, K2a2a, T1, U2c, U6a7a2, N1b1a3, M29a, M2b, M20, C7, S1, Z1	El Sidron 1253, Feldhofer 1, Feldhofer 2, Vindija 33.16, Vindija 33.17, Vindija 33.19, Vindija 33.25
185	L0a1b1	Altai, Mezmaiskaya 1
189	L0a1b1, L5a1a, W1, C4	Altai Neanderthal, El Sidron 1253, Feldhofer 1, Feldhofer 2, Mezmaiskaya 1, Vindija 33.16, Vindija 33.17, Vindija 33.19, Vindija 33.25
195	L2a1f, L4a1, L5a1a, L6a, W1, K, J2a2a, M2b	Altai Neanderthal
247	L0a1b1, L1c3a, L5a1a	Altai Neanderthal, El Sidron 1253, Feldhofer 1, Feldhofer 2, Mezmaiskaya 1, Vindija 33.16, Vindija 33.17, Vindija 33.19, Vindija 33.25
709	L5a1a, L6a, W1, K2a2a, T1, Y1, N2a, G1a1, S1	Altai Neanderthal, El Sidron 1253, Feldhofer 1, Feldhofer 2, Mezmaiskaya 1, Vindija 33.16, Vindija 33.17, Vindija 33.19, Vindija 33.25
769	L0a1b1, L1c3a, L2a1f, L4a1, L5a1a, L6a	Altai Neanderthal, El Sidron 1253, Feldhofer 1, Feldhofer 2, Mezmaiskaya 1, Vindija 33.16, Vindija 33.17, Vindija 33.19, Vindija 33.25
825	L0a1b1, L1c3a, L5a1a	Altai Neanderthal, El Sidron 1253, Feldhofer 1, Feldhofer 2, Mezmaiskaya 1, Vindija 33.16, Vindija 33.17, Vindija 33.19, Vindija 33.25
827	R0a1a3, B2	Altai Neanderthal, El Sidron 1253, Feldhofer 1, Feldhofer 2, Mezmaiskaya 1, Vindija 33.16, Vindija 33.17, Vindija 33.19, Vindija 33.25
1018	L0a1b1, L1c3a, L2a1f, L4a1, L5a1a, L6a	Altai Neanderthal, El Sidron 1253, Feldhofer 1, Feldhofer 2, Mezmaiskaya 1, Vindija 33.16, Vindija 33.17, Vindija 33.19, Vindija 33.25
2706	U6a7a2, rCRS, H1a1, H3, H15	Altai Neanderthal, El Sidron 1253, Feldhofer 1, Feldhofer 2, Mezmaiskaya 1, Vindija 33.16, Vindija 33.17, Vindija 33.19, Vindija 33.25
2758	L0a1b1, L1c3a	Altai Neanderthal, Mezmaiskaya 1
2885	L0a1b1, L1c3a	Altai Neanderthal, El Sidron 1253, Feldhofer 1, Feldhofer 2, Mezmaiskaya 1, Vindija 33.16, Vindija 33.17, Vindija 33.19, Vindija 33.25
3010	H1a1, J1c, D4	Altai Neanderthal, El Sidron 1253, Feldhofer 1, Feldhofer 2, Mezmaiskaya 1, Vindija 33.16, Vindija 33.17, Vindija 33.19, Vindija 33.25
3594	L0a1b1, L1c3a, L2a1f, L5a1a, L6a	Altai Neanderthal, El Sidron 1253, Feldhofer 1, Feldhofer 2, Mezmaiskaya 1, Vindija 33.16, Vindija 33.17, Vindija 33.19, Vindija 33.25
4104	L0a1b1, L1c3a, L2a1f, L5a1a	Altai Neanderthal, El Sidron 1253, Feldhofer 1, Feldhofer 2, Mezmaiskaya 1, Vindija 33.16, Vindija 33.17, Vindija 33.19, Vindija 33.25
4312	L0a1b1	Altai Neanderthal, El Sidron 1253, Feldhofer 1, Feldhofer 2, Mezmaiskaya 1, Vindija 33.16, Vindija 33.17, Vindija 33.19, Vindija 33.25
4688	L3d3b	Feldhofer 1, Vindija 33.25
5460	L0a1b1, L4a1, W1, Q1	Altai Neanderthal, El Sidron 1253, Feldhofer 1, Feldhofer 2, Mezmaiskaya 1, Vindija 33.16, Vindija 33.17, Vindija 33.19, Vindija 33.25
5471	U6a7a2, N1b1a3	Altai Neanderthal, El Sidron 1253, Feldhofer 1, Feldhofer 2, Mezmaiskaya 1, Vindija 33.16, Vindija 33.17, Vindija 33.19, Vindija 33.25
5821	C7	Altai Neanderthal, El Sidron 1253, Feldhofer 1, Feldhofer 2, Mezmaiskaya 1, Vindija 33.16, Vindija 33.17, Vindija 33.19, Vindija 33.25
6962	F1a1	Mezmaiskaya 1

7146	L0a1b1, Lc3a	Altai Neanderthal, Mezmaiskaya 1
7256	L0a1b1, L1c3a, L2a1f, L5a1a, L6a	Altai Neanderthal, El Sidron 1253, Feldhofer 1, Feldhofer 2, Mezmaiskaya 1, Vindija 33.16, Vindija 33.17, Vindija 33.19, Vindija 33.25
7424	L3d3b, L5a1a	Altai Neanderthal, El Sidron 1253, Feldhofer 1, Feldhofer 2, Mezmaiskaya 1, Vindija 33.16, Vindija 33.17, Vindija 33.19, Vindija 33.25
7521	L0a1b1, L1c3a, L2a1f, L5a1a, R0a	Altai Neanderthal, El Sidron 1253, Feldhofer 1, Feldhofer 2, Mezmaiskaya 1, Vindija 33.16, Vindija 33.17, Vindija 33.19, Vindija 33.25
7650	R0a	Altai Neanderthal, El Sidron 1253, Feldhofer 1, Feldhofer 2, Vindija 33.16, Vindija 33.17, Vindija 33.19, Vindija 33.25
8386	J2a2a	Altai Neanderthal, El Sidron 1253, Feldhofer 1, Feldhofer 2, Mezmaiskaya 1, Vindija 33.16, Vindija 33.17, Vindija 33.19, Vindija 33.25
8468	L0a1b1, Lc3a	Altai Neanderthal, El Sidron 1253, Feldhofer 1, Feldhofer 2, Mezmaiskaya 1, Vindija 33.16, Vindija 33.17, Vindija 33.19, Vindija 33.25
8655	L0a1b1, L1c3a, L5a1a	Altai Neanderthal, El Sidron 1253, Feldhofer 1, Feldhofer 2, Mezmaiskaya 1, Vindija 33.16, Vindija 33.17, Vindija 33.19, Vindija 33.25
8701	L0a1b1, L1c3a, L2a1f, L3d3b, L4a1, L5a1a, L6a, M29a, M2b, M3b, M8a1, M9a, M20, G1a1, E1, Q1, Z1, C1a, C4, C7	Altai Neanderthal, El Sidron 1253, Feldhofer 1, Feldhofer 2, Mezmaiskaya 1, Vindija 33.16, Vindija 33.17, Vindija 33.19, Vindija 33.25
9053	F1a1	Altai Neanderthal, El Sidron 1253, Feldhofer 1, Feldhofer 2, Mezmaiskaya 1, Vindija 33.16, Vindija 33.17, Vindija 33.19, Vindija 33.25
9090	Z1	Altai Neanderthal
9540	L0a1b1, L2a1f, L3d3b, L4a1, L5a1a, L6a, C1a, C4, C7, D4, E1, G1a1, M20, M29a, M2b, M3b, M8a1, M9a, Q1, Z1	Altai Neanderthal, El Sidron 1253, Feldhofer 1, Feldhofer 2, Mezmaiskaya 1, Vindija 33.16, Vindija 33.17, Vindija 33.19, Vindija 33.25
9755	L0a1b1	Altai Neanderthal, El Sidron 1253, Feldhofer 1, Feldhofer 2, Mezmaiskaya 1, Vindija 33.16, Vindija 33.17, Vindija 33.19, Vindija 33.25
10310	F1a1, A	Altai Neanderthal, El Sidron 1253, Feldhofer 1, Feldhofer 2, Mezmaiskaya 1, Vindija 33.16, Vindija 33.17, Vindija 33.19, Vindija 33.25
10373	L4a1	Altai Neanderthal, El Sidron 1253, Feldhofer 1, Feldhofer 2, Mezmaiskaya 1, Vindija 33.16, Vindija 33.17, Vindija 33.19, Vindija 33.25
10398	L0a1b1, L1c3a, L2a1f, L3d3b, L4a1, L5a1a, L6a, J1c, I1, J2a2a, K, K1, C1a, C4, C7, D4, E1, G1a1, M20, M29a, M2b, M3b, M8a1, M9a, Q1, Y1, Z1	Altai Neanderthal, El Sidron 1253, Feldhofer 1, Feldhofer 2, Mezmaiskaya 1, Vindija 33.16, Vindija 33.17, Vindija 33.19, Vindija 33.25
10586	L1c3a	Feldhofer 2
10664	L0a1b1	Altai Neanderthal, El Sidron 1253, Feldhofer 1, Feldhofer 2, Mezmaiskaya 1, Vindija 33.16, Vindija 33.17, Vindija 33.19, Vindija 33.25
10688	L0a1b1, L1c3a, L5a1a	Altai Neanderthal, El Sidron 1253, Feldhofer 1, Feldhofer 2, Mezmaiskaya 1, Vindija 33.16, Vindija 33.17, Vindija 33.19, Vindija 33.25
10810	L0a1b1, L1c3a, L5a1a	Altai Neanderthal, El Sidron 1253, Feldhofer 1, Feldhofer 2, Mezmaiskaya 1, Vindija 33.16, Vindija 33.17, Vindija 33.19, Vindija 33.25
10873	L0a1b1, L1c3a, L2a1f, L3d3b, L4a1, L5a1a, L6a, Q1, Z1, C1a, C4, C7, M29a, M2b, M3b, M8a1, M9a, M20, G1a1, E1, D4	Altai Neanderthal, El Sidron 1253, Feldhofer 1, Feldhofer 2, Mezmaiskaya 1, Vindija 33.16, Vindija 33.17, Vindija 33.19, Vindija 33.25

10915	L0a1b1	Altai Neanderthal, El Sidron 1253, Feldhofer 1, Feldhofer 2, Mezmaiskaya 1, Vindija 33.16, Vindija 33.17, Vindija 33.19, Vindija 33.25
11914	L0a1b1, L2a1f, K2a2a, C1a, C4, C7, M20	Altai Neanderthal, El Sidron 1253, Feldhofer 1, Feldhofer 2, Vindija 33.16, Vindija 33.17, Vindija 33.19, Vindija 33.25
12366	M29a	Altai Neanderthal, El Sidron 1253, Feldhofer 1, Feldhofer 2, Mezmaiskaya 1, Vindija 33.16, Vindija 33.17, Vindija 33.19, Vindija 33.25
12406	F1a1	Altai Neanderthal, El Sidron 1253, Feldhofer 1, Feldhofer 2, Mezmaiskaya 1, Vindija 33.16, Vindija 33.17, Vindija 33.19, Vindija 33.25
12705	L0a1b1, L1c3a, L2a1f, L3d3b, L4a1, L5a1a, L6a, W1, X1a, I1, X3, A, C1a, C4, C7, D4, E1, G1a1, M20, M29a, M2b, M3b, M8a1, M9a, N1b1a3, N2a, N9a1, O, Q1, S1, Y1, Z1	Altai Neanderthal, El Sidron 1253, Feldhofer 1, Feldhofer 2, Mezmaiskaya 1, Vindija 33.16, Vindija 33.17, Vindija 33.19, Vindija 33.25
12810	L1c3a, K1	Mezmaiskaya 1
13105	L0a1b1, L1c3a, L3d3b, L5a1a, V2	Altai Neanderthal, El Sidron 1253, Feldhofer 1, Feldhofer 2, Mezmaiskaya 1, Vindija 33.16, Vindija 33.17, Vindija 33.19, Vindija 33.25
13276	L0a1b1	Altai Neanderthal, El Sidron 1253, Feldhofer 1, Feldhofer 2, Mezmaiskaya 1, Vindija 33.16, Vindija 33.17, Vindija 33.19, Vindija 33.25
14178	L1c3a, Y1	Altai Neanderthal, El Sidron 1253, Feldhofer 1, Feldhofer 2, Mezmaiskaya 1, Vindija 33.16, Vindija 33.17, Vindija 33.19, Vindija 33.25
14560	L1c3a, X3	Altai Neanderthal, El Sidron 1253, Feldhofer 1, Feldhofer 2, Mezmaiskaya 1, Vindija 33.16, Vindija 33.17, Vindija 33.19, Vindija 33.25
15043	U6a7a2, U1a1d, I1, C1a, C4, C7, D4, E1, G1a1, M20, M29a, M2b, M3b, M8a1, M9a, Q1, Z1	Altai Neanderthal, El Sidron 1253, Feldhofer 1, Feldhofer 2, Mezmaiskaya 1, Vindija 33.16, Vindija 33.17, Vindija 33.19, Vindija 33.25
15148	U1a1d, M8a1	Altai Neanderthal, El Sidron 1253, Feldhofer 1, Feldhofer 2, Mezmaiskaya 1, Vindija 33.16, Vindija 33.17, Vindija 33.19, Vindija 33.25
15244	L6a, V2	Altai Neanderthal
15301	L2a1f, L3d3b, L4a1, L6a, K C1a, C4, C7, D4, E1, G1a1, M20, M29a, M2b, M3b, M8a1, M9a, Q1, Z1	Altai Neanderthal, El Sidron 1253, Feldhofer 1, Feldhofer 2, Mezmaiskaya 1, Vindija 33.16, Vindija 33.17, Vindija 33.19, Vindija 33.25
15355	M8a1, B2	Altai Neanderthal, El Sidron 1253, Feldhofer 1, Feldhofer 2, Mezmaiskaya 1, Vindija 33.16, Vindija 33.17, Vindija 33.19, Vindija 33.25
16086	M8a1, M20	Mezmaiskaya 1
16093	L4a1, R0a, K1, H3, T1, A, C1a	Feldhofer 1, Vindija 33.25
16148	L0a1b1, L5a1a, Q1	Altai Neanderthal, El Sidron 1253, Feldhofer 1, Feldhofer 2, Mezmaiskaya 1, Vindija 33.16, Vindija 33.17, Vindija 33.19, Vindija 33.25
16170	U2c	Altai Neanderthal, El Sidron 1253, Feldhofer 1, Feldhofer 2, Mezmaiskaya 1, Vindija 33.16, Vindija 33.17, Vindija 33.19, Vindija 33.25
16182	X1a, X3, U1a1d, M2b, M29a, O	Feldhofer 2, Mezmaiskaya 1, Vindija 33.19
16183	X1a, X3, U1a1d, B2, M2b, M29a, O	El Sidron 1253, Feldhofer 1, Feldhofer 2, Mezmaiskaya 1, Vindija 33.16, Vindija 33.17, Vindija 33.19, Vindija 33.25
16209	H1a1, S1, M20	Altai Neanderthal, El Sidron 1253, Feldhofer 1, Feldhofer 2, Mezmaiskaya 1, Vindija 33.16, Vindija 33.17, Vindija 33.19, Vindija 33.25
16223	L0a1b1, L1c3a, L2a1f, L3d3b, L4a1, L5a1a, L6a, X3, I1, W1, X1a, A, C1a, C4, C7, D4, E1, G1a1, M20, M29a, M2b, M3b, M8a1, M9a, N1b1a3, N2a, N9a1, O, Q1, S1, Z1	Altai Neanderthal, El Sidron 1253, Feldhofer 1, Feldhofer 2, Mezmaiskaya 1, Vindija 33.16, Vindija 33.17, Vindija 33.19, Vindija 33.25

16230	L0a1b1	Altai Neanderthal, El Sidron 1253, Feldhofer 1, Feldhofer 2, Mezmaiskaya 1, Vindija 33.16, Vindija 33.17, Vindija 33.19, Vindija 33.25
16234	U2c, M9a	Altai Neanderthal, El Sidron 1253, Feldhofer 1, Feldhofer 2, Mezmaiskaya 1, Vindija 33.16, Vindija 33.17, Vindija 33.19, Vindija 33.25
16278	L0a1b1, L1c3a, L2a1f, L5a1a, L6a, X3, U2c, P2	Altai Neanderthal, El Sidron 1253, Feldhofer 1, Feldhofer 2, Mezmaiskaya 1, Vindija 33.16, Vindija 33.17, Vindija 33.19, Vindija 33.25
16298	V1a, V2, Z1, M8a1, C1a, C4, C7	Altai Neanderthal
16311	L0a1b1, L1c3a, L4a1, I1, L5a1a, L6a, V1a, K, K1, K2a2a, R1a, O, Q1, M29a	Altai Neanderthal, El Sidron 1253, Feldhofer 1, Feldhofer 2, Mezmaiskaya 1, Vindija 33.16, Vindija 33.17, Vindija 33.19, Vindija 33.25
16320	L0a1b1, M2b	Altai Neanderthal, El Sidron 1253, Feldhofer 1, Feldhofer 2, Mezmaiskaya 1, Vindija 33.16, Vindija 33.17, Vindija 33.19, Vindija 33.25
16362	L4a1, L5a1a, L6a, V2, K1, U1a1d, I1, R0a, R0a1a3, R1a, G1a1, E1, D4, M9a, A	Altai Neanderthal, El Sidron 1253, Feldhofer 1, Feldhofer 2, Mezmaiskaya 1, Vindija 33.16, Vindija 33.17, Vindija 33.19, Vindija 33.25
16519	L1c3a, L2a1f, L6a, H15, H1a1, H3, I1, K, K1, K2a2a, R0a1a3, R1a, U2c, V1a, W1, X1a, X3, B2, C4, C7, E1, F1a1, G1a1, M20, M29a, M2b, M3b, N1b1a3, N2a, O, P2	Altai Neanderthal, El Sidron 1253, Feldhofer 1, Feldhofer 2, Mezmaiskaya 1, Vindija 33.16, Vindija 33.17, Vindija 33.19, Vindija 33.25
